# Screening and Analysis of the Potential Bioactive Components of *Poria cocos* (Schw.) Wolf by HPLC and HPLC-MS^n^ with the Aid of Chemometrics

**DOI:** 10.3390/molecules21020227

**Published:** 2016-02-18

**Authors:** Ling-Fang Wu, Kun-Feng Wang, Xin Mao, Wen-Yi Liang, Wen-Jing Chen, Shi Li, Qi Qi, Ya-Ping Cui, Lan-Zhen Zhang

**Affiliations:** School of Chinese Materia Medica, Beijing University of Chinese Medicine, Beijing 100102, China; fanglingwu@163.com (L.-F.W.); fengqiwu66463@sina.com (K.-F.W.); mxmaoxin@126.com (X.M.); lwy1054289310@163.com (W.-Y.L.); sdcwjing@163.com (W.-J.C.); lishi816@126.com (S.L.); cici_jiayou@163.com (Q.Q.); 20150931830@bucm.edu.cn (Y.-P.C.)

**Keywords:** triterpene acids, fingerprints, cluster analysis, principal component analysis

## Abstract

The aim of the present study was to establish a new method based on Similarity Analysis (SA), Cluster Analysis (CA) and Principal Component Analysis (PCA) to determine the quality of different samples of *Poria cocos* (Schw.) Wolf obtained from Yunnan, Hubei, Guizhou, Fujian, Henan, Guangxi, Anhui and Sichuan in China. For this purpose 15 samples from the different habitats were analyzed by HPLC-PAD and HPLC-MS^n^. Twenty-three compounds were detected by HPLC-MS^n^, of which twenty compounds were tentatively identified by comparing their retention times and mass spectrometry data with that of reference compounds and reviewing the literature. The characteristic fragmentations were summarized. 3-*epi*-Dehydrotumulosic acid (**F13**), 3-oxo-16α,25-dihydroxylanosta-7,9(11),24(31)-trien-21-oic acid (**F4**), 3-oxo-6,16α-dihydroxylanosta-7,9(11),24(31)-trien-21-oic acid (**F7**) and dehydropachymic acid (**F15**) were deemed to be suitable marker compounds to distinguish between samples of different quality according to CA and PCA. This study provides helpful chemical information for further anti-tumor activity and active mechanism research on *P. cocos*. The results proved that fingerprint combined with a chemometric approach is a simple, rapid and effective method for the quality discrimination of *P*. *cocos*.

## 1. Introduction

*Poria cocos* (Schw.) Wolf is a saprophytic fungus that grows on diverse species of *Pinus*. Its sclerotium, called Fu-Ling or hoelen, is used in traditional Chinese and Japanese medicine for its diuretic, sedative, and tonic effects. *Poria cocos* (Schw.) Wolf is widely used in Yunnan, Guizhou, Hubei, Anhui, Fujian, Sichuan and Guangxi provinces in China, Modern medical research has indicated that *P. cocos* had comprehensive biological activities, such as antitumor [1,2,3,4,5,6,7,8,9,10,11], anti-inflammatory [12,13,14,15,16,17,18], immune-modulating [19,20], liver protecting [21,22] and so on, but particularly antitumor activity. *Poria cocos* (Schw.) Wolf contains a variety of triterpene acids found to be the bioactive components [2,3,4,5,6,7,8], for example pachymic acid, tumulosic acid, polyporenic acid C, dehydroeburicoic acid, dehydropachymic acid and so on. The type and content of triterpene acids reflect the quality of *P. cocos* so triterpene acids could be used as marker components to evaluate the quality of *P. cocos*.

The therapeutic effects of traditional Chinese medicines (TCMs) are based on the complex interactions of complicated chemical constituents as a whole system, so methods are needed in order to control the quality of the complex system. In this case, HPLC fingerprints of key components provide a new approach for quality control of traditional Chinese medicines. There are many studies about fingerprints analysis combined with chemometrics for the quality control of traditional Chinese medicines and to find the bioactive components [23,24,25,26,27].

Some studies on the fingerprints of *Poria cocos* (Schw.) Wolf have been reported [28,29,30,31], but in those reports only a few compounds were identified by HPLC-MS^n^ and the characteristic fragmentations were not summarized. No marker compounds were found from cluster analysis (CA) and principal component analysis (PCA).

In the present study, nineteen common peaks and four other peaks which have not been detected using HPLC were identified by high–resolution liquid mass spectrometry. To the best of our knowledge, this is the first time that so many compounds were identified and their characteristic fragmentations summarized. We also found for the first time that 3-*epi*-dehydrotumulosic acid (**F13**), 3-oxo-16α,25-dihydroxylanosta-7,9(11),24(31)-trien-21-oic acid (**F4**), 3-oxo-6,16α-dihydroxylanosta-7,9(11),24(31)-trien-21-oic acid (**F7**) and dehydropachymic acid (**F15**) might be suitable marker compounds to distinguish between *P.* *cocos* samples with different quality according to CA and PCA. This study provides helpful chemical information for further anti-tumor activity and active mechanism research on *P.* *cocos*. The method developed in our study also provides a scientific foundation for the origin discrimination and quality control of *P.* *cocos*.

## 2. Results and Discussion

### 2.1. Validation of the Method

The relative retention time, relative peak area and similarities were used to evaluate the quality of the fingerprints. Dehydrotumulosic acid (peak 8) which is a large single peak in the middle of the chromatogram, was assigned as the reference peak to calculate relative retention times and relative peak areas.

The precision was determined by replicate injection with the same sample solution six consecutive times. The RSDs of relative retention time and relative peak area of the common peaks were all below 0.87% and 1.47%, respectively; the similarities of different chromatograms were all above 0.995.

The repeatability was evaluated by the analysis of six prepared samples. The RSDs of relative retention time and relative retention time of the common peaks were all below 1.59% and 1.97%, respectively; the similarities of different chromatograms were all above 0.995.

Stability testing was performed with one sample over 24 h. The RSDs of relative retention time and relative retention time of the common peaks were all below 0.96% and 1.98%; the similarities of different chromatograms were all 1.000. All these results indicated that the samples remained stable during the testing period and the conditions for the fingerprint analysis were satisfactory.

### 2.2. Similarity Analysis (SA)

The chromatographic profile must be representative of all the samples and have the features of integrity and fuzziness. By analyzing the mutual pattern of chromatograms, the identification and authentication of the samples can be conducted well even if the amounts of some chemical constituents are different from the others.

**Figure 1:**
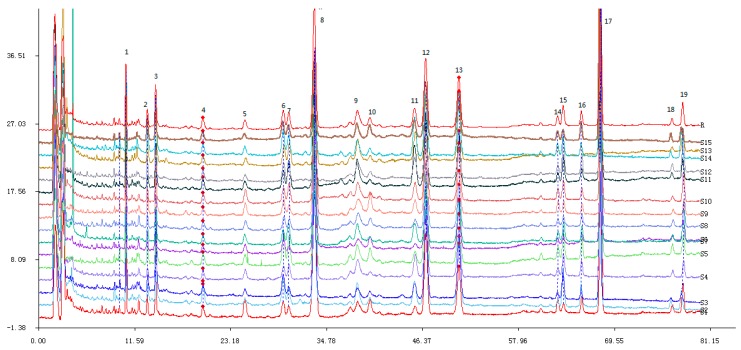
Overlaid HPLC chromatograms of samples from No. S1–S15. The common pattern (marked R) was obtained by using Similarity Evaluation System (SES) for Chromatographic Fingerprint of TCM.

Fifteen batches of samples from different habitats were determined and the chromatograms analyzed by SES to generate a common pattern R (Figure 1). SES for Chromatographic Fingerprint was performed to calculate the similarities of different chromatograms compared to the common pattern. The results are shown in Table 1.

The conclusion can be drawn from the results that the similarities of different chromatograms compared to the common pattern are all above 0.900 except for samples S4 (0.880), S6 (0.885), S7 (0.872), S11 (0.860) and S13 (0.875), which indicates that the chemical constituents of different samples are not influenced highly by sources. The common pattern is a very positive identification for the samples of *P. cocos.*

### 2.3. Identification of the Compounds Present

**Figure 2:**
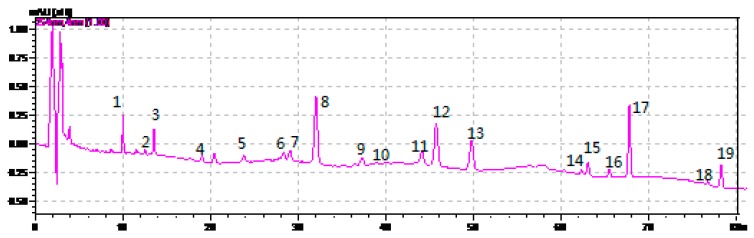
HPLC chromatograms of *P. cocos*.

**Figure 3:**
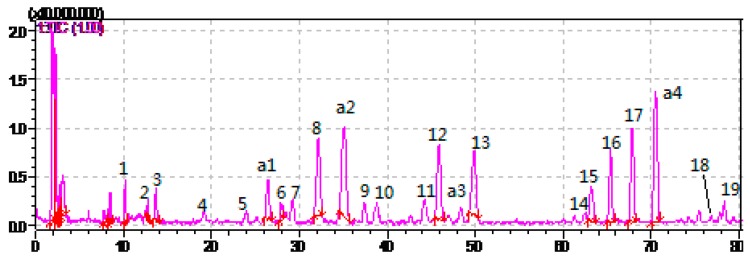
Positive mode of the HPLC-MS^n^ chromatograms of *P. cocos*.

**Figure 4:**
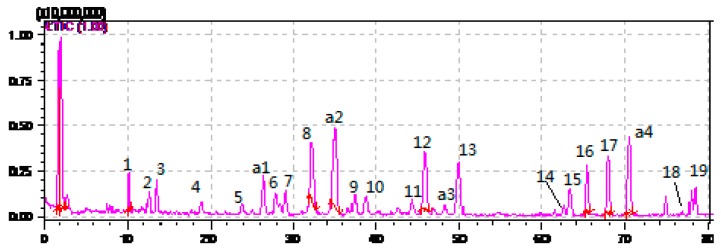
Negative mode of the HPLC-MS^n^ chromatograms of *P. cocos*.

**Figure 5:**
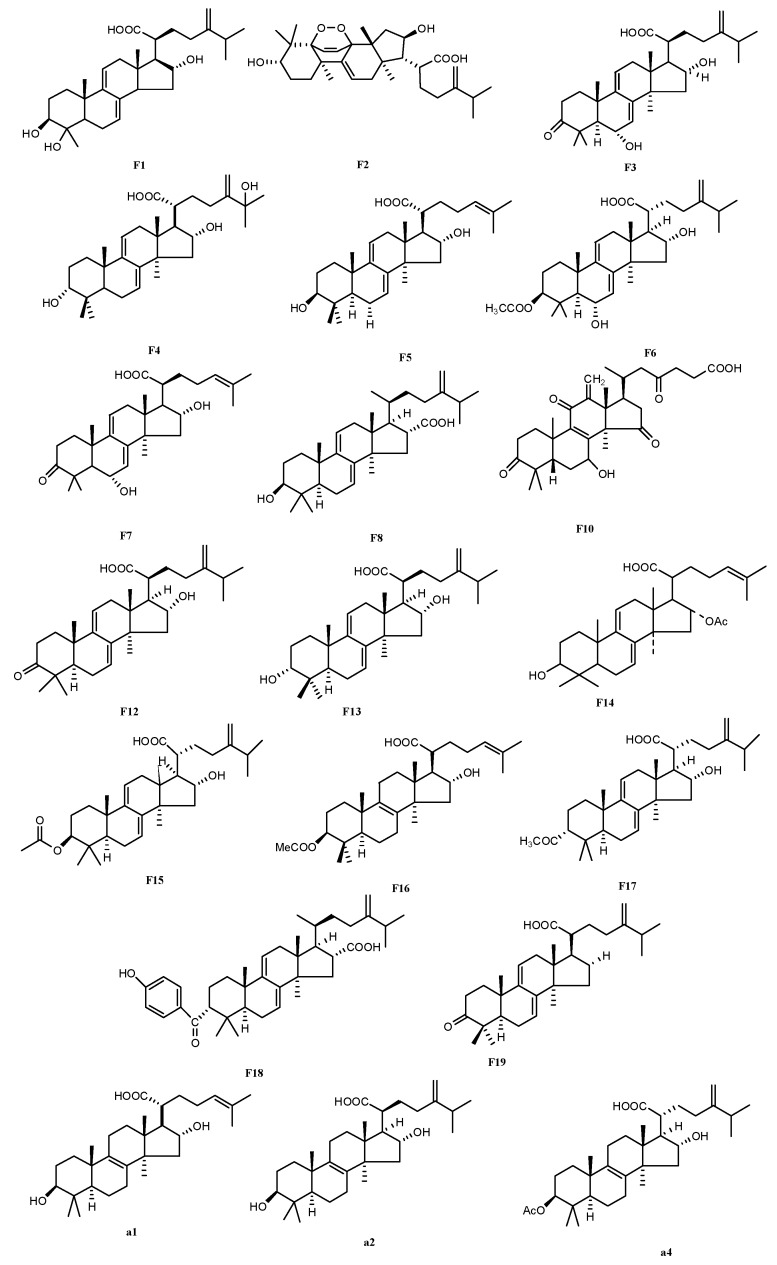
The chemical structures of the identified compounds.

HPLC-ESI-MS^n^ method was employed to identify the components in *P*. *cocos* (Figure 2, Figure 3 and Figure 4) Molecular weight and fragmentation information (Table 2) were obtained. The possible structures of these 19 common peaks and four other peaks a1, a2, a3 and a4 were deduced as it shown in Figure 5. Under the optimized MS conditions, positive mode and negative mode were used to identify the peaks.

As shown in Table 2, in the positive mode ESI-MS^1^ spectra, the [M + H]^+^ and [M − H_2_O + H]^+^ ions were observed for all 23 compounds except for compound **F11**. The [M + Na]^+^ ions were seen for all the compounds except for compounds **F1**–**F3**, **F6**, **F11**, **F16** and **F19**. The [M − 2H_2_O + H]^+^ ions were seen for all the compounds except for compounds **F2**, **F4**, **F11**, **F12**, **F15**, **F17**, **F18** and **F19**. Compounds **F6**, **F14**–**F17** and **a4** showed the corresponding [M − H_2_O − CH_3_COOH + H]^+^ ions. [M + K]^+^ ions were observed for compounds **F5**, **F8**, **F13**, **a1**, **a2** and **a4**. The [M − HCOOH + H]^+^ ions were found for compounds **F8**, **F12**, **F13**, **F15** and **F17**. [M − 2H_2_O − RDA fragmentation of B ring + H]^+^ ions were found for compounds **F7**, **F10**, **F14** and **a3**. The [M − CH_3_COOH + H]^+^ ions were observed for compounds **F15**−**F17** and **a4**. Compounds **F8**, **F13** and **a3** presented [M − H_2_O − side chain on D ring + H]^+^ ions, while **F3** and **a1** showed [M − CH_3_ − RDA fragmentation of B ring + H]^+^ ions. [M − 2H_2_O − CH_3_COOH + H]^+^ ions were found for compounds **F6** and **F16**. [M − RDA fragmentation of B ring + H]^+^ ions were found for compounds **F6** and **F10**. Compounds **F7** and **F12** presented [M − H_2_O − RDA fragmentation of B ring + H]^+^ ions, while **F7** and **F10** displayed [M − CH_3_ − side chain on D ring + H]^+^ions and **F7** also displayed an [M − 2CH_3_ − side chain on D ring + H]^+^ ion. The [M − CO_2_ + H]^+^ ion was observed compounds **a1** and **F16**.Compound **F9** presented [M − CH_3_ − H_2_O − side chain on D ring + H]^+^, [M − 2CH_3_ − H_2_O − HCOOH + H]^+^ and [M − 2H_2_O − HCOOH − RDA fragmentation of B ring + H]^+^ ions, while **F10** had [M − 3H_2_O + H]^+^, [M − 2H_2_O − HCOOH + H]^+^ and [M − H_2_O − CH_3_ − side chain on D ring + H]^+^ ones. The [M − side chain on D ring + H]^+^ ion was seen in the spectrum of compound **F12**. A [M − HCOOH − RDA fragmentation of B ring + H]^+^ ion was seen for compound **a3**. Compound **a2**, on the other hand, displayed [M − H_2_O − HCO−Ar−OH + H]^+^, [M − 2H_2_O–HCO–Ar–OH + H]^+^ and [M − CH_3_ − HCOOH–HCO–Ar–OH + H]^+^ ions, whereas **a1** had an [M − CH_4_ + H]^+^ ion.

In the ESI-MS^2^ spectra, all 23 compounds except for compound **F11** displayed the corresponding [M − H_2_O + H]^+^ ions.[M − 2H_2_O + H]^+^ ions were found for all the compounds except for compounds **F2**, **F4**, **F5**, **F11**, **F15**, **F17**, **F18** and **a4**. Compounds **F2**, **F6**–**F9**, **F12**–**F14** and **a3** showed [M − (H_2_O + side chain on D ring) + H]^+^ ions and compounds **F5**, **F14**−**F17** and **a4** showed [M − (H_2_O + CH_3_COOH) + H]^+^ ones. [M − side chain on D ring + H]^+^ ions were seen for compounds**F4**, **F19**, while **F6** and **F19** had [M − RDA fragmentation of B ring + H]^+^ ions. The [M − RDA fragmentation of B ring − 2H_2_O + H]^+^ ion was observed for **F4** and **F9**. Only **F2** had [M − 3H_2_O + H]^+^ and [M − 2H_2_O − HCOOH + H]^+^ ions. The ions [M − 2CH_3_ + H]^+^, [M − 2CH_3_ − H_2_O + H]^+^, [M − 2CH_3_ − RDA fragmentation of B ring + H]^+^, [M − RDA fragmentation of B ring − 2H_2_O − HCOOH + H]^+^ and [M − side chain on D ring − 2CH_3_ + H]^+^ were only found for compound **F4**, and only compound **F5** had a [M − (2H_2_O + CH_3_COOH) + H]^+^ ion. Other ions seen in only one compound were [M − side chain on D ring − 2H_2_O − CH_3_COOH + H]^+^ in compound **F6** and [M − HCOOH + H]^+^ for **F19**.

In the ESI-MS^3^ spectra all 23 compounds except for compounds **F2**, **F11** and **F18** displayed [MS^2^ − H_2_O + H]^+^ ions, while compounds **F1**, **F3**, **F6**–**F9** and **a3** also showed a [MS^2^ − 2H_2_O + H]^+^ ion. All 23 compounds except for **F4** and **F11** had [MS^2^ − side chain on D ring + H]^+^ ions. The [MS^2^ − side chain on D ring − H_2_O + H]^+^ ion was observed in the spectra of compounds **F1**−**F3**, **F5**, **F6**, **F8**, **F13** and **a1**–**a4** and **F1**, **F3**, **F6**–**F9** and **a3** showed a [MS^2^ − 2H_2_O + H]^+^ ion. [MS^2^ − HCOOH + H]^+^ ions were seen for compounds **F3**, **F6**, **F9**, **F12**, **F16**, **a3** and **a4**, while **F14**−**F17** and **a4** showed both [MS^2^ − CH_3_COOH + H]^+^ and [MS^2^ − CH_3_COOH − side chain on D ring + H]^+^ ions. Compounds **F4**, **F8**, **F13** and **F18** produced [MS^2^ − 2CH_3_ + H]^+^ ions and **F8**, **F13** and **F19** had an ion corresponding to a [MS^2^ − RDA fragmentation of B ring + H]^+^ species. The [MS^2^ − CH_3_COOH − H_2_O + H]^+^ ion was noted for compounds **F16** and **a4**. The latter compound also had a [MS^2^ − CH_3_ + H]^+^ ion. [MS^2^ − HCOOH − H_2_O + H]^+^ ions were found for compounds **F1** and **F3**. Compound **F5** displayed a [MS^2^ − CH_3_ − H_2_O + H]^+^ ion while compound **F4** showed [MS^2^ − RDA fragmentation of B ring − H_2_O + H]^+^ and [MS^2^ − RDA fragmentation of B ring − H_2_O − 2CH_3_ + H]^+^ ones and compound **F9** showed a [MS^2^ − RDA fragmentation of B ring − 2H_2_O + H]^+^ ion. The [MS^2^ − HCO–Ar–OH + H]^+^, [MS^2^ − HCOOH − (HCO–Ar–OH) + H]^+^ and [MS^2^ − side chain on D ring − (HCO–Ar–OH) + H]^+^ ions were observed in the spectrum of compound **F18**.

In the negative mode ESI-MS^1^ spectra, the [M − H]^−^ ions were found for all 23 compounds except for compound **F11**. Compound **F6** had [M − CH_3_COOH − H]^−^ and [M − side chain on D ring − H]^−^ ion. Compound **F8** presented [M − 2CH_3_ − side chain on D ring − H]^−^, [M − 4CH_3_ − HCOOH − RDA fragmentation of B ring − H]^−^, [M − H_2_O − H]^−^ and [M − 3CH_3_ − H]^−^ ions, while compound **F10** had [M − CH_4_ − H]^−^ and [M − CH_4_ − 2H_2_O − HCOOH − RDA fragmentation of B ring − H]^−^ ions. A [M − K]^−^ ion was found for compound **F12**. [M − 2CH_4_ − side chain on D ring − H]^−^, [M − 2H_2_O − H]^−^ and [M − CO − H]^−^ ions were only seen for compounds **F13**, **F16** or**F19**, respectively.

In the ESI-MS^2^ spectra, [M − H_2_O − H]^−^ ions were found for compounds **F1**, **F3**, **F7**–**F9** and **F13**, while [M − CH_4_ − HCOOH − H]^−^ ions were seen for compounds **F1**, **F10**, **F16** and **a1**–**a4**. Meanwhile, compounds **F8**, **F12**, **F13, F16**, **a3** and **a4** showed a [M − HCOOH − H]^−^ ion and **F6**, **F14**–**F17** formed a [M − CH_3_COOH − H]^−^ ion. The [M − CO_2_ − H]^−^ ion was seen for compounds **F5**, **F10**, **F12** and **F14** and **F15**, **F17** and **F19** displayed a [M − CH_4_ − H]^−^ ion. The [M − 2CH_4_ − HCOOH − H]^−^ ion was observed for compounds **F3**, **F12** and **a3**. The latter compound, **F8** and **F13** displayed [M − 2CH_4_ − side chain on D ring − H]^−^ ions. [M − 2CH_4_ − H]^−^ ions were found for compounds **F1** and **F2**, while **F1** and **F19** had [M − H_2_O − side chain on D ring − H]^−^ ions. Compounds **F3** and **a3** showed a [M − 2CH_4_ − H_2_O − HCOOH − H]^−^ ion, **F8** and **F13** had a [M − CH_4_ − side chain on D ring − H]^−^ ion and **F9** and **a2** had [M − 3CH_3_ − H]^−^ ion. [M − H_2_O − CH_3_COOH − H]^−^ and [M − H_2_O − CH_4_ − CH_3_COOH − H]^−^ ions were found for compounds **F15** and **F17**. Compound **a3** had [M − 2H_2_O − 2CH_4_ − HCOOH − H]^−^ and [M − 3CH_4_ − HCOOH − H]^−^ ions, whereas **a4** showed [M − 2H_2_O − CH_3_COOH − H]^−^ and [M − CH_4_ − HCOOH − CH_3_COOH − H]^−^ ions and **a2** showed a [M − 3CH_4_ − H]^−^ ion. Compound **F10** presented a [M − CH_4_ − 2H_2_O − HCOOH − RDA fragmentation of B ring − H]^−^ ion while compound **F1** had a [M − CH_4_ − H_2_O − HCOOH − H]^−^ one. A [M − H_2_O − HCOOH − H]^−^ ions was observed for compound **F3**. Compound **F4** showed a [M − 2CH_3_ − H]^−^ ion. The [M − CH_4_ − HCOOH − RDA fragmentation of B ring − H]^−^ ion was observed for compound **F9** and compound **F12** presented a peak for a [M − 2CH_4_ − RDA fragmentation of B ring − H]^−^ ion. The [M − 2H_2_O − CH_4_ − CO_2_ − CH_3_COOH − H]^−^ ion was observed in the spectrum of compound **F14** and an [M − HCO−Ar−OH −H]^−^ ion was found for compound **F18**.

In the ESI-MS^3^ spectra, [MS^2^ − CH_4_ − H]^−^ ions were seen for compounds **F1**, **F3**, **F5**, **F14** and **a2**, while compounds **F16** and **a4** presented [MS^2^ − CH_3_COOH − H]^−^ ions and compound **2** showed a [MS^2^ − CH_3_ − H]^−^ ion. Compound **F4** showed [MS^2^ − HCOOH − H]^−^ and [MS^2^ − 2CH_4_ − HCOOH − H]^−^ ions. Finally, compound **F12** showed the corresponding [MS^2^ − H_2_O − H]^−^ ion.

### 2.4. Cluster Analysis (CA)

Cluster Analysis is a multivariate analysis technique that is used to sort samples into groups. It is widely applied for fingerprint analysis, because it is a nonparametric data interpretation method and simple to use. CA provides a visual representation of complex data. Average linkage between groups was applied, and Pearson correlation was selected as a measurement. The method can classify different herbs by measuring the peak areas from their corresponding HPLC fingerprints. The common characteristic peaks, which were calculated by the Similarity Evaluation System, were selected for the CA. Cluster analysis of *P. cocos* samples was performed based on the relative peak areas of all 19 common peaks.

**Figure 6:**
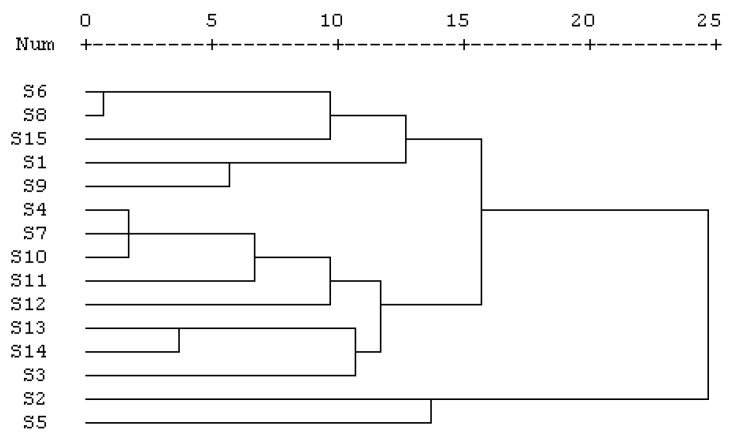
Results of cluster analysis of 15 samples.

The results of CA are shown in Figure 6, where the quality characteristics are revealed more clearly. The cluster analysis results show that the samples could be divided into three quality clusters. Among them, Cluster I includes the samples S6, S8, S15, S1 and S9, Cluster III includes S2, S5 and the others are in Cluster II. All the compounds in Cluster III had much higher concentrations than the other two clusters.

Cluster I was distinguished as it contains less 3-*epi*-dehydrotumulosic acid (**F13**), 3-oxo-16α,25-dihydroxylanosta-7,9(11),24(31)-trien-21-oic acid (**F4**), 3-oxo-6,16α-dihydroxylanosta-7,9(11),24(31)-trien-21-oic acid (**F7**), dehydropachymic acid (**F15**), Unknown **F9**, and Unknown **F11** than Clusters II and III. The low concentration of these six compounds in Cluster I may be due to the poor herb quality of *P.* *cocos*. This indicated that these compounds could be used as marker compounds to distinguish the *P. cocos* samples with different quality. The results of CA could be validated against each other and provided more references for the quality evaluation of *P. cocos*.

### 2.5. Principal Components Analysis (PCA)

**Figure 7:**
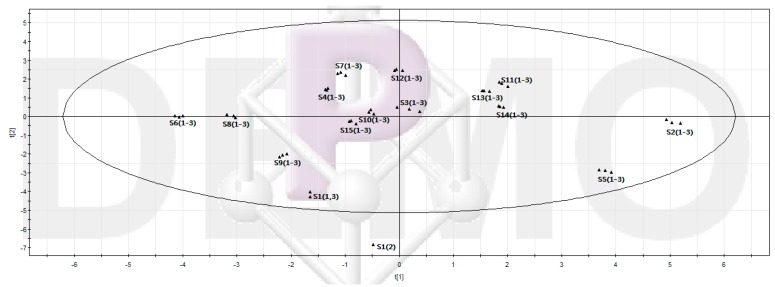
PCA scores plots of the sample from different regions with 95% confidence ellipses.

To evaluate the variations in quality of the 15 samples, PCA was carried out with the relative amounts of each identified component. The contents of 19 fingerprint peaks were applied to evaluate the sample variations. Figure 7 shows the score plots obtained by PCA. The first six principal components accounted for 89.329% of the total variance. Examination of the score plots indicates that the main components responsible for the separation were 3-*epi*-dehydrotumulosic acid (**F13**), 6α-hydroxyldehydropachymic acid (**F6**), 24(31)-trien-21-oic acid (**F4**), 24(31)-trien-21-oic acid (**F7**), 3-oxo-6,16α-dihydroxylanosta-7, 9 (**F15**), 29-hydroxydehydrotumulosic acid (**F1**), dehydropachymic acid (**F12**), as shown in Table 3. These components were deemed to be the marker compounds of sample variation. This result is in accord with the one obtained from the cluster analysis (CA). The combination of PCA and CA was thus a useful tool for quality control and evaluation of *P. cocos*.

## 3. Experimental Section

### 3.1. Samples and Reagents

Fifteen *P. cocos* samples were purchased from different regions of China and were authenticated by Professor Chun-Sheng Liu (School of Chinese Materia Medica, Beijing University of Chinese Medicine, Beijing, China). The samples were harvested between July and September. The samples were processed as follows: the sediment was removed after them digging up, and the material was piled to “sweat”, spread out until the surface was dry, then “sweated” again. This was repeated several times until the surface of the samples was wrinkled and the water in the sample was almost dissipated. Samples were then dried in the shade, peeled and cut into cubes. The surface of the blocks is white or faint red in color. Each sample (three replicates) was placed in a dark and dry environment. The regions where the 15 samples were obtained are shown in Table 4. Pachymic acid (Batch number: 130306, purity ≥ 98%) and dehydroeburicoic acid (Batch number: 131027, purity ≥ 98%) were obtained from Chengdu MUST BioTechnology Co., Ltd. (Chengdu, China); HPLC grade acetonitrile and acetic acid were obtained from Fisher (Waltham, MA, USA); distilled water was bought from Watsons (Beijing, China) and was filtered through a 0.45 µm membrane (Dikma, Beijing, China) prior to use. All other reagents were of analytical grade.

### 3.2. Sample Preparation

#### 3.2.1. Preparation of Reference Substance

Stock solutions of individual reference substance were prepared by dissolving each compound in 50% methanol at a concentration of 212 µg·mL^−1^ for pachymic acid and 22.9 µg·mL^−1^ for dehydroeburicoic acid. Both solutions were stored at approximately 4 °C.

#### 3.2.2. Preparation of Sample Solution

Dried powder of *P. cocos* from different regions (1 g) was accurately weighed out and transferred into a 100 mL conical flask. Methanol (10 mL) was added to the flask and the flask with the methanol and powder was accurately weighed and placed in an ultrasonic extraction device and extracted for 30 min. The flask was weighed again and methanol was added to make up the weight. The solution was filtered through a 0.45 µm membrane filter for fingerprint analysis.

### 3.3. Apparatus and Parameters

A Waters Alliance HPLC 2695 series instrument (Waters, Manchester, UK) was used to perform the high performance liquid chromatography (HPLC) analysis. Mobile phase: A (acetonitrile); B (H_2_O:CH_3_COOH, 100:0.2, *v*/*v*). Column: Diamansil™ C18 (250 × 4.6 mm, 5 μm), maintained at 30 °C with flow rate of 1.0 mL·min. The detection wavelength was set at 254 nm for acquiring chromatograms. The injection volume was 20 µL. Gradient elution procedure: 0 min (45% A) → 8 min (55% A) → 22 min (55% A) → 55 min (65% A) → 56 min (70% A) → 80 min (90% A).

The LCMS-IT-TOF instrument (Shimadzu, Kyoto, Japan) was equipped with an ESI source used in positive and negative ionization mode. The interface and MS parameters were as follows: nebulizer pressure, 100 kPa; dry gas, N_2_ (1.5 L/min); drying gas temperature, 200 °C; spray capillary voltage, 4000 V; scan range, *m*/*z* 100–1500.

## 4. Conclusions

The therapeutic effects of traditional Chinese medicines (TCM) are based on the complex interactions of complicated chemical constituents as a whole system. HPLC and HPLC-MS^n^ fingerprint analysis combined with chemometrics were employed to study the complex *P*. *cocos* system. Triterpenoid acids were the most important chemical components in the samples, which had a variety of potential biological activities, according to previous extensive phytochemical and pharmacological studies. The qualitative analysis and quantification of triterpenoid acids can better reflect the therapeutic effects and quality of *P.* *cocos*. The chromatographic method is predominantly to control the quality and stability of the complex system. This study provided a systematic method for the quality control of *P*. *cocos* by HPLC fingerprinting and the HPLC-MS^n^ evaluation system based on Similarity Analysis (SA), Cluster Analysis (CA) and Principal Component Analysis (PCA). As a result, a common mutual pattern was established by determining and comparing the fingerprints of 15 samples of *P*. *cocos* from different regions. Twenty-three compounds were detected by HPLC-MS^n^, of which twenty were tentatively identified by comparing their retention times, and mass spectrometry data with that of reference compounds and literature data. The characteristic fragmentations were summarized. 3-*epi*-Dehydrotumulosic acid (**F13**), 3-oxo-16α,25-dihydroxy-lanosta-7,9(11),24(31)-trien-21-oic acid (**F4**), 3-oxo-6,16α-dihydroxylanosta-7,9(11),24(31)-trien-21-oic acid (**F7**) and dehydropachymic acid (**F15**) were deemed to be the markers to distinguish between *P*. *cocos* samples of different quality. The proposed method can be used to improve the quality control of *P*. *cocos*, thus ensuring the effectiveness of *Poria* herbs. There are still three peaks—**F9**, **F11** and **a3**—which were not identified by HPLC-MS^n^, of which **F9** and **F11** were used as marker compounds to distinguish the *P. cocos* of different quality. These two components require further study.

## Figures and Tables

**Table 1:** The results of similarities of the chromatograms from different origins.

NO.	S1	S2	S3	S4	S5	S6	S7	S8	S9	S10	S11	S12	S13	S14	S15	R
S1	1.000	0.848	0.860	0.897	0.927	0.800	0.717	0.962	0.944	0.953	0.806	0.828	0.819	0.843	0.804	0.935
S2	0.848	1.000	0.944	0.803	0.953	0.874	0.884	0.911	0.941	0.930	0.831	0.923	0.863	0.982	0.934	0.973
S3	0.860	0.944	1.000	0.730	0.912	0.844	0.841	0.914	0.911	0.940	0.707	0.920	0.916	0.914	0.797	0.943
S4	0.897	0.803	0.730	1.000	0.921	0.660	0.641	0.879	0.866	0.886	0.933	0.767	0.723	0.799	0.852	0.880
S5	0.927	0.953	0.912	0.921	1.000	0.821	0.815	0.952	0.962	0.967	0.902	0.921	0.869	0.942	0.931	0.985
S6	0.800	0.874	0.844	0.660	0.821	1.000	0.902	0.876	0.922	0.832	0.607	0.812	0.649	0.856	0.818	0.885
S7	0.717	0.884	0.841	0.641	0.815	0.902	1.000	0.804	0.865	0.811	0.632	0.843	0.721	0.870	0.827	0.872
S8	0.962	0.911	0.914	0.879	0.952	0.876	0.804	1.000	0.974	0.984	0.814	0.905	0.813	0.896	0.853	0.971
S9	0.944	0.941	0.911	0.866	0.962	0.922	0.865	0.974	1.000	0.959	0.820	0.911	0.809	0.933	0.918	0.985
S10	0.953	0.930	0.940	0.886	0.967	0.832	0.811	0.984	0.959	1.000	0.835	0.937	0.891	0.913	0.858	0.980
S11	0.806	0.831	0.707	0.933	0.902	0.607	0.632	0.814	0.820	0.835	1.000	0.772	0.716	0.856	0.900	0.860
S12	0.828	0.923	0.920	0.767	0.921	0.812	0.843	0.905	0.911	0.937	0.772	1.000	0.875	0.910	0.854	0.940
S13	0.819	0.863	0.916	0.723	0.869	0.649	0.721	0.813	0.809	0.891	0.716	0.875	1.000	0.853	0.737	0.875
S14	0.843	0.982	0.914	0.799	0.942	0.856	0.870	0.896	0.933	0.913	0.856	0.910	0.853	1.000	0.945	0.965
S15	0.804	0.934	0.797	0.852	0.931	0.818	0.827	0.853	0.918	0.858	0.900	0.854	0.737	0.945	1.000	0.927
R	0.935	0.973	0.943	0.880	0.985	0.885	0.872	0.971	0.985	0.980	0.860	0.940	0.875	0.965	0.927	1.000

**Table 2:** The HPLC-MS^n^ data and compound names of the 20 peaks.

Peak No.	*t*_R_ (min)	[M − H]^−^ [M + H]^+^	Negative Mode	Positive Mode	Identification
**F1**	10.228	499.3346 501.3562	**MS^1^**: 499.3346 [M – H]^−^ **MS^2^**: 499.3346→481.3221 [M – 18(H_2_O) − H]^−^, 467.3075 [M − 32(2CH_4_) − H]^−^, 437.2931 [M − 62(CH_4_ + HCOOH) − H]^−^, 419.2964 [M − 80(CH_4_ + HCOOH + H_2_O) − H]^−^, 325.2526 [M − 174(H_2_O + side chainon D ring) − H]^−^ **MS^3^**: 419.2964→403.2698 [M − 80(H_2_O + CH_4_ + HCOOH) − 16(CH_4_) − H]^−^	**MS^1^**: 501.3562 [M + H]^+^, 483.3447 [M − 18(H_2_O) + H]^+^, 465.3384 [M − 36(2H_2_O) + H]^+^ **MS^2^**: 501.3562→483.3460 [M − 18(H_2_O) + H]^+^, 465.3330 [M − 36(2H_2_O) + H]^+^ **MS^3^**: 483.3460→465.3324 [M − 18(H_2_O) − 18(H_2_O) + H]^+^, 447.3232 [M − 18(H_2_O) − 36(2H_2_O) + H]^+^, 419.3319 [M − 18(H_2_O) − 64(H_2_O + HCOOH) + H]^+^, 327.2338 [M − 18(H_2_O) − 156(side chain on D ring) + H]^+^, 309.2208 [M − 18(H_2_O) − 174(H_2_O + side chain on D ring) + H]^+^, 465.3324→447.3118 [M − 36(2H_2_O) − 18(H_2_O) + H]^+^, 309.2139 [M − 36(2H_2_O) − 156(side chain on D ring) + H]^+^, 291.2027 [M − 36(2H_2_O) − 174(H_2_O + side chain on D ring) + H]^+^	29-Hydroxy-dehydrotumulosic acid [32]
**F2**	12.637	513.3213 515.3352	**MS^1^**: 513.3213 [M − H]^−^ **MS^2^**: 513.3213→481.3303 [M − 32(2CH_4_) − H]^−^ **MS^3^**: 481.3303→466.3146 [M − 32(2CH_4_) − 15(CH_3_) − H]^−^	**MS^1^**: 515.3352 [M + H]^+^, 497.3238 [M − 18(H_2_O) + H]^+^ **MS^2^**: 515.3352→497.3235 [M − 18(H_2_O) +H]^+^, 461.3047 [M − 54(3H_2_O) + H]^+^, 433.3021 [M − 82(2H_2_O + HCOOH) + H]^+^, 341.2130 [M − 174(H_2_O + side chain on D ring) + H]^+^ **MS^3^**: 497.3238→341.2081 [M − 18(H_2_O) − 156(side chain on D ring) + H]^+^, 323.1985 [M − 18(H_2_O) − 174(H_2_O + side chain on D ring) + H]^+^	5α, 8α-Peroxy-dehydrotumulosic acid [5]
**F3**	13.665	497.3254 499.3394	**MS^1^**: 497.3254 [M − H]^−^ **MS^2^**: 497.3254→479.3096 [M − 18(H_2_O) − H]^−^, 435.3142 [M − 64(H_2_O + HCOOH) − H]^−^, 419.2947 [M − 78(2CH_4_ + HCOOH) − H]^−^, 401.2782 [M − 96(2CH_4_ + H_2_O + HCOOH) − H]^−^ **MS^3^**: 419.2947→403.2698 [M − 78(2CH_4_ + HCOOH) − 16(CH_4_) − H]^−^	**MS^1^**: 499.3494 [M + H]^+^, 481.3307 [M − 18(H_2_O) + H]^+^, 463.3196 [M − 36(2H_2_O) + H]^+^, 346.3312 [M − 153(CH_3_ + RDA fragmentation of B ring) + H]^+^ **MS^2^**: 499.3394→481.3306 [M − 18(H_2_O) + H]^+^, 463.3205 [M − 36(2H_2_O) + H]^+^ **MS^3^**: 481.3306→463.3220 [M − 18(H_2_O) − 18(H_2_O) + H]^+^, 445.3217 [M − 18(H_2_O) − 36(2H_2_O) + H]^+^, 417.3181 [M − 18(H_2_O) − 64(H_2_O + HCOOH) + H]^+^, 325.2182 [M − 18(H_2_O) − 156(side chain on D ring) + H]^+^ or [M − 18(H_2_O) − 156(H_2_O + RDA fragmentation of B ring) + H]^+^, 307.2059 [M − 18(H_2_O) − 174(H_2_O + side chain on D ring) + H]^+^, 463.3205→445.3125 [M − 36(2H_2_O) − 18(H_2_O) + H]^+^, 417.3209 [M − 36(2H_2_O) − 46(HCOOH) + H]^+^, 307.1993 [M − 36(2H_2_O) − 156(side chain on D ring) + H]^+^	6α-Hydroxy-polyporenic acid C [33]
**F4**	19.150	497.3287 499.3436	**MS^1^**: 497.3287 [M − H]^−^ **MS^2^**: 497.3287→467.3141 [M − 30(2CH_3_) − H]^−^ **MS^3^**: 467.3141→421.3067 [M − 30(2CH_3_) − 46 (HCOOH) − H]^−^, 389.2857 [M − 30(2CH_3_) − 78 (2CH_4_ + HCOOH) − H]^−^	**MS^1^**: 499.3436 [M + H]^+^, 521.3314 [M + Na]^+^, 481.3343 [M − 18(H_2_O) + H]^+^ **MS^2^**: 499.3436→481.3343 [M − 18(H_2_O) + H]^+^, 469.3290 [M − 30(2CH_3_) + H]^+^, 451.3264 [M − 48(2CH_3_ + H_2_O) + H]^+^, 330.6835 [M − 68(2CH_3_ + RDA fragmentation of B ring) + H]^+^, 327.1625 [M − 172(side chain on D ring) + H]^+^, 325.2160 [M − 174(2H_2_O + RDA fragmentation of B ring) + H]^+^, 297.1598 [M − 202(2CH_3_ + side chain on D ring) + H]^+^, 279.1605 [M − 220(2H_2_O + HCOOH + RDA fragmentation of B ring) + H]^+^ **MS^3^**: 481.3433→463.3205 [M − 18(H_2_O) − 18(H_2_O) + H] ^+^, 451.3202 [M − 18(H_2_O) − 30(2CH_3_) + H]^+^, 325.2160 [M − 18(H_2_O) − 156(H_2_O + RDA fragmentation of B ring) + H]^+^, 295.2101 [M − 18(H_2_O) − 186(2CH_3_ + H_2_O + RDA fragmentation of B ring) + H]^+^, 451.3264→295.2045 [M − 48(2CH_3_ + H_2_O) − 156(H_2_O + RDA fragmentation of B ring) + H]^+^	3-oxo-16α,25-dihydroxylanosta-7,9(11),24(31)-trien-21-oic acid
**F5**	23.845	469.3329 471.3508	**MS^1^**: 469.3329 [M − H]^−^ **MS^2^**: 469.3329→425.3429 [M − 44(CO_2_) − H]^−^ **MS^3^**: 425.3429→409.3112 [M − 44(CO_2_) − 16(CH_4_) − H]^−^	**MS^1^**: 471.3508 [M + H]^+^, 509.3063 [M + K]^+^, 493.3397 [M + Na]^+^, 453.3371 [M − 18(H_2_O) + H]^+^, 435.3267 [M − 36(2H_2_O) + H]^+^ **MS^2^**: 471.3508→453.3350 [M − 18(H_2_O) + H]^+^ **MS^3^**: 453.3350→435.3268 [M − 18(H_2_O) − 18(H_2_O) + H]^+^, 311.2349 [M − 18(H_2_O) − 142(side chain on D ring) + H]^+^, 293.2289 [M − 18(H_2_O) − 160(H_2_O + side chain on D ring) + H]^+^, 311.2349→293.2229 [M − 18(H_2_O) − 142(side chain on D ring) − 18(H_2_O) + H]^+^, 278.2023 [M − 18(H_2_O) − 142(side chain on D ring) − 33(H_2_O + CH_3_) + H]^+^	3β,16α-Dihydroxy-lanosta-7,9(11),24-trien-21-oic acid [34]
**F6**	28.422	541.3569 543.3700	**MS^1^**: 541.3569 [M − H]^−^, 481.3308 [M − 60(CH_3_COOH) − H]^−^, 384.9361 [M − 156(side chain on D ring) − H]^−^ **MS^2^**: 541.3569→481.3293 [M − 60(CH_3_COOH) − H]^−^	**MS^1^**: 543.3700 [M + H]^+^, 525.3596 [M − 18(H_2_O) + H]^+^, 507.3346 [M − 36(2H_2_O) + H]^+^, 465.3378 [M − 78(H_2_O + CH_3_COOH) + H]^+^, 447.3277 [M − 96(2H_2_O + CH_3_COOH) + H]^+^, 361.6931 [M − 182(RDA fragmentation of B ring) + H]^+^ **MS^2^**: 543.3700→525.3521 [M − 18(H_2_O) + H]^+^, 507.3462 [M − 36(2H_2_O) + H]^+^, 465.3363 [M − 78(H_2_O + CH_3_COOH) + H]^+^, 447.3277 [M − 96(2H_2_O + CH_3_COOH) + H]^+^, 369.2441 [M − 174(H_2_O + side chain on D ring) + H]^+^, 361.6931 [M − 182(RDA fragmentation of B ring) + H]^+^, 291.2117 [M − 18(H_2_O) − 234(H_2_O + side chain on D ring + CH_3_COOH) + H]^+^ **MS^3^**: 465.3378→447.3228 [M − 78(H_2_O + CH_3_COOH) − 18(H_2_O) + H]^+^, 429.3027 [M − 78(H_2_O + CH_3_COOH) − 36(2H_2_O) + H]^+^, 419.3290 [M − 78(H_2_O + CH_3_COOH) − 46(HCOOH) + H]^+^, 309.2194 [M − 78(H_2_O + CH_3_COOH) − 156(side chain on D ring) + H]^+^, 291.2111 [M − 78(H_2_O + CH_3_COOH) − 174(H_2_O + side chain on D ring) + H]^+^, 447.3277→291.2103 [M − 96(2H_2_O+CH_3_COOH) − 156(side chain on D ring) + H]^+^	6α-Hydroxy-dehydropachymic acid [34]
**F7**	29.043	483.2973 485.3333	**MS^1^**: 483.2973 [M − H]^−^ **MS^2^**: 483.2973→465.2966 [M − 18(H_2_O) − H]^−^	**MS^1^**: 485.3332 [M + H]^+^, 507.3094 [M + Na]^+^, 467.3190 [M − 18(H_2_O) + H]^+^, 449.3071 [M − 36(2H_2_O) + H]^+^, 328.9961 [M − 156(H_2_O + RDA fragmentation of B ring) + H]^+^, 326.0151 [M − 158(CH_4_ + side chain on D ring) + H]^+^, 311.1511 [M − 174(2CH_4_ + side chain on D ring) + H]^+^ or [M − 174(2H_2_O + RDA fragmentation of B ring) + H]^+^ **MS^2^**: 485.3333→467.3242 [M − 18(H_2_O) + H] ^+^, 449.3070 [M − 36(2H_2_O) + H]^+^, 325.2146 [M − 160(H_2_O + side chain on D ring) + H]^+^ **MS^3^**: 467.3242→449.3048 [M − 18(H_2_O) − 18(H_2_O) + H]^+^, 431.2976 [M − 18(H_2_O) − 36(2H_2_O) + H]^+^, 325.2218 [M − 18(H_2_O) − 142(side chain on D ring) + H]^+^, 449.3070→ 431.3046 [M − 36(2H_2_O) − 18(H_2_O) + H]^+^, 307.2038 [M − 36(H_2_O) − 142(side chain on D ring) + H]^+^	3-oxo-6,16α-dihydroxylanosta-7,9(11),24(31)-trien-21-oic acid [35]
**F8**	32.068	483.3425 485.3602	**MS^1^**: 483.3425 [M − H]^−^, 295.2370 [M − 188(2CH_4_ + side chain on D ring) − H]^−^ **MS^2^**: 483.3425→465.2955 [M − 18(H_2_O) − H]^−^, 437.3440 [M − 46(HCOOH) − H]^−^, 311.1998 [M − 172(CH_4_+side chain on D ring) − H]^−^, 295.2036 [M − 188(2CH_4_ + side chain on D ring) − H]^−^	**MS^1^**: 485.3602 [M + H]^+^, 523.3198 [M + K]^+^, 507.3451 [M + Na]^+^, 467.3518 [M − 18(H_2_O) + H]^+^, 449.3387 [M − 36(2H_2_O) + H]^+^, 439.3618 [M − 46(HCOOH) + H]^+^, 311.1665 [M − 174(H_2_O + side chain on D ring) + H]^+^ **MS^2^**: 485.3602→467.3512 [M − 18(H_2_O) + H]^+^, 449.3496 [M − 36(2H_2_O) + H]^+^, 311.2428 [M − 174(H_2_O + side chain on D ring) + H]^+^ **MS^3^**: 467.3512→449.3409 [M − 18(H_2_O) − 18(H_2_O) + H]^+^, 431.2786 [M − 18(H_2_O) − 36(2H_2_O) + H]^+,^ 327.2293 [M − 18(H_2_O) − 140(RDA fragmentation of B ring) + H]^+^, 311.2351 [M − 18(H_2_O) − 156(side chain on D ring) + H]^+^, 293.2248 [M − 18(H_2_O) − 174(H_2_O + side chain on D ring) + H]^+^, 311.2428→293.2308 [M − 174(H_2_O + side chain on D ring) − 18(H_2_O) + H]^+^, 281.6503 [M − 174(H_2_O + side chain on D ring) − 30(2CH_3_) + H]^+^	Dehydrotumulosic acid [32]
**F9**	37.338	497.3263 499.3444	**MS^1^**: 497.3263 [M − H]^−^, 479.3193 [M − 18(H_2_O) − H]^−^, 452.9247 [M − 45(3CH_3_) − H]^−^, 248.9602 [M − 248 (4CH_4_ + HCOOH + RDA fragmentation of B ring) − H]^−^ **MS^2^**: 497.3263→479.3161 [M − 18(H_2_O) − H]^−^, 452.9247 [M − 45(3CH_3_) − H]^−^, 249.9602 [M − 248(4CH_4_ + HCOOH + RDA fragmentation of B ring) − H]^−^	**MS^1^**: 521.3305 [M + Na]^+^, 499.3444 [M + H]^+^, 481.3334 [M − 18(H_2_O) + H]^+^, 463.3219 [M − 36(2H_2_O) + H]^+^, 405.2614 [M − 93(2CH_3_ + H_2_O + HCOOH) + H]^+^, 310.1678 [M − 189(CH_3_ + H_2_O + side chain on D ring) + H]^+^, 279.1589 [M − 220(2H_2_O + HCOOH + RDA fragmentation of B ring) + H]^+^ **MS^2^**: 499.3444→481.3324 [M − 18(H_2_O) + H]^+^, 463.3205 [M − 36(2H_2_O) + H]^+^, 325.2130 [M − 174(H_2_O + side chain on D ring) + H]^+^ or [M − 174(2H_2_O + RDA fragmentation of B ring) + H]^+^ **MS^3^**: 481.3324→463.3215 [M − 18(H_2_O) − 18(H_2_O) + H]^+^, 445.3115 [M − 18(H_2_O) − 36(2H_2_O) + H]^+^, 325.2132 [M − 18(H_2_O) − 156(side chain on D ring) + H]^+^ or [M − 174(2H_2_O + RDA fragmentation of B ring) + H]^+^, 463.3205→445.3046 [M − 36(2H_2_O) − 18(H_2_O) + H]^+^, 417.3143 [M − 36(2H_2_O) − 46(HCOOH) + H]^+^, 307.2058 [M − 36(2H_2_O) − 156(side chain on D ring) + H]^+^	Unknown
**F10**	38.513	485.3269 487.3491	**MS^1^**: 485.3269 [M − H]^−^, 469.3311 [M − 16(CH_4_) − H]^−^, 248.9582 [M − 236(CH_4_ + 2H_2_O + HCOOH + RDA fragmentation of B ring) − H]^−^ **MS^2^**: 485.3269→441.3391 [M − 44(CO_2_) − H]^−^, 423.3255 [M − 62(CH_4_ + HCOOH) − H]^−^, 248.9582 [M − 236(CH_4_ + 2H_2_O + HCOOH + RDA fragmentation of B ring − H)]^−^	**MS^1^**: 509.3283 [M + Na]^+^, 487.3491 [M + H]^+^, 469.3318 [M − 18(H_2_O) + H]^+^, 451.3180 [M − 36(2H_2_O) + H]^+^, 433.3214 [M − 54(3H_2_O) + H]^+^, 405.2659 [M − 82(2H_2_O + HCOOH) + H]^+^, 348.9844 [M − 138(RDA fragmentation of B ring) + H]^+^, 327.0051 [M − 18(H_2_O) − 142(side chain on D ring) + H]^+^, 313.1531 [M − 174(2H_2_O + RDA fragmentation of B ring) + H]^+^, 312.1531 [M − 18(H_2_O) − 15(CH_3_) − 142(side chain on D ring) + H]^+^ **MS^2^**: 487.3491→469.3290 [M − 18(H_2_O) + H]^+^, 451.3169 [M − 36(2H_2_O) + H]^+^	3-oxo-6,16α-Dihydroxytra-metenolic acid [36]
**F11**	44.363	−−−−−	−−−−−−	−−−−−−	
**F12**	45.727	481.3333 483.3448	**MS^1^**: 481.3333 [M − H]^−^ **MS^2^**: 481.3333→437.3338 [M − 44(CO_2_) − H]^−^, 435.3259 [M − 46(HCOOH) − H]^−^, 403.2999 [M − 78(2CH_4_ + HCOOH) − H]^−^, 311.2015 [M − 170(2CH_4_ + RDA fragmentation of B ring) − H]^−^ **MS^3^**: 311.2015→293.2008 [M − 170(2CH_4_ + RDA fragmentation of B ring) − 18(H_2_O) − H]^−^	**MS^1^**: 483.3463 [M + H]^+^, 505.3322 [M + Na]^+^, 465.3360 [M − 18(H_2_O) + H]^+^, 437.3412 [M − 46(HCOOH) + H]^+^, 327.0080 [M − 156(side chain on D ring) + H]^+^ or [M − 156(H_2_O + RDA fragmentation of B ring) + H]^+^ **MS^2^**: 483.3448→465.3357 [M − 18(H_2_O) + H]^+^, 447.2191 [M − 36(2H_2_O) + H]^+^, 309.2130 [M − 174(H_2_O + side chain on D ring) + H]^+^ **MS^3^**: 465.3357→447.3255 [M − 18(H_2_O) − 18(H_2_O) + H]^+^, 419.3318 [M − 18(H_2_O) − 46(HCOOH) + H]^+^, 309.2194 [M − 18(H_2_O) − 156(side chain on D ring) + H]^+^	Polyporenic acid C [32]
**F13**	49.785	483.3478 485.3613	**MS^1^**: 483.3425 [M − H]^−^, 295.2370 [M − 188(2CH_4_ + side chain on D ring) − H]^−^ **MS^2^**: 483.3425→465.2955 [M − 18(H_2_O) − H]^−^, 437.3440 [M − 46(HCOOH) − H]^−^, 311.1998 [M − 172(CH_4_ + side chain on D ring) − H]^−^, 295.2036 [M − 188(2CH_4_ + side chain on D ring) − H]^−^	**MS^1^**: 485.3602 [M + H]^+^, 523.3198 [M + K]^+^, 507.3451 [M + Na]^+^, 467.3518 [M − 18(H_2_O) + H]^+^, 449.3387 [M − 36(2H_2_O) + H]^+^, 439.3618 [M − 46(HCOOH) + H]^+^, 311.1665 [M − 174(H_2_O+side chain on D ring) + H]^+^ **MS^2^**: 485.3602→467.3512 [M − 18(H_2_O) + H]^+^, 449.3496 [M − 36(2H_2_O) + H]^+^, 311.2428 [M − 174(H_2_O + side chain on D ring) + H]^+^ **MS^3^**: 467.3512→449.3409 [M − 18(H_2_O) − 18(H_2_O) + H]^+^, 431.2786 [M − 18(H_2_O) − 36(2H_2_O) + H]^+^, 327.2293 [M − 18(H_2_O) − 140(RDA fragmentation of B ring) + H]^+^, 311.2351 [M − 18(H_2_O) − 156(side chain on D ring) + H]^+^, 293.2248 [M − 18(H_2_O) − 174(H_2_O + side chain on D ring) + H]^+^, 311.2428→293.2308 [M − 174(H_2_O + side chain on D ring) − 18(H_2_O) + H]^+^, 281.6503 [M − 174(H_2_O + side chain on D ring) − 30(2CH_3_) + H]^+^	3-*epi*-Dehydrotumulosic acid [36]
**F14**	62.492	511.3436 513.3544	**MS^1^**: 511.3433 [M − H]^−^ **MS^2^**: 511.3436→467.3499 [M − 44(CO_2_) − H]^−^, 451.3122 [M − 60(CH_3_COOH) − H]^−^, 355.2211 [M − 156(2H_2_O + CO_2_ + CH_4_ + CH_3_COOH) − H]^−^ **MS^3^**: 467.3499→451.3222 [M − 44(CO_2_) − 16(CH_4_) − H]^−^	**MS^1^**: 513.3544 [M + H]^+^, 495.3478 [M − 18(H_2_O) + H]^+^, 477.3357 [M − 36(2H_2_O) + H]^+^, 435.3200 [M − 18(H_2_O) − 60(CH_3_COOH) + H]^+^, 337.6933 [M − 176(2H_2_O + RDA fragmentation of B ring) + H]^+^ **MS^2^**: 513.3544→495.3446 [M − 18(H_2_O) + H]^+^, 477.3298 [M − 36(2H_2_O) + H]^+^, 435.3185 [M − 78(H_2_O + CH_3_COOH) + H]^+^, 353.2502 [M − 160(H_2_O + side chain on D ring) + H]^+^ **MS^3^**: 495.3446→435.3266 [M − 18(H_2_O) − 60(CH_3_COOH) + H]^+^, 353.2445 [M − 18(H_2_O) − 142(side chain on D ring) + H]^+^, 293.2276 [M − 18(H_2_O) − 202(CH_3_COOH + side chain on D ring) + H]^+^, 435.3185→293.2244 [M − 78(H_2_O + CH_3_COOH) − 142(side chain on D ring) + H]^+^	3β-Hydroxy-16α-acetoxylanosta-7,9(11),24-trien-21-oic acid [36]
**F15**	63.130	525.3603 527.3735	**MS^1^**: 525.3581 [M − H]^−^ **MS^2^**: 525.3603→509.3196 [M − 16(CH_4_) − H]^−^, 465.3379 [M − 60(CH_3_COOH) − H]^−^, 447.3200 [M − 78(H_2_O + CH_3_COOH) − H]^−^, 432.3020 [M − 93(CH_3_ + H_2_O + CH_3_COOH) − H]^−^	**MS^1^**: 527.3735 [M + H]^+^, 549.3522 [M + Na]^+^, 509.3624 [M − 18(H_2_O) + H]^+^, 481.3769 [M − 46(HCOOH) + H]^+^, 467.3539 [M − 60(CH_3_COOH) + H]^+^, 449.3400 [M − 78(H_2_O + CH_3_COOH) + H]^+^ **MS^2^**: 527.3735→509.3624 [M − 18(H_2_O) + H]^+^, 449.3465 [M − 78(H_2_O + CH_3_COOH) + H]^+^ **MS^3^**: 509.3624→491.3414 [M − 18(H_2_O) − 18(H_2_O) + H]^+^, 449.3399 [M − 18(H_2_O) − 60(CH_3_COOH) + H]^+^, 353.2453 [M − 18(H_2_O) − 156(side chain on D ring) + H]^+^, 293.2240 [M − 18 (H_2_O) − 216(CH_3_COOH + side chain on D ring) + H]^+^, 449.3465→293.2249 [M − 78(H_2_O + CH_3_COOH) − 156(side chain on D ring) + H]^+^	Dehydropachymic acid [32]
**F16**	65.458	513.3579 515.3762	**MS^1^**: 513.3580 [M − H]^−^, 487.3071 [M − 36(2H_2_O) − H]^−^ **MS^2^**: 513.3579→467.3514 [M − 46(HCOOH) − H]^−^, 453.3324 [M − 60(CH_3_COOH) − H]^−^, 451.3184 [M − 46(HCOOH) − 16(CH_4_) − H]^−^ **MS^3^**: 451.3184→391.3126 [M − 46(HCOOH) − 16(CH_4_) − 60(CH_3_COOH) − H]^−^	**MS^1^**: 515.3761 [M + H]^+^, 497.3646 [M − 18(H_2_O) + H]^+^, 479.3530 [M − 36(2H_2_O) + H]^+^, 471.3044 [M − 44(CO_2_) + H]^+^, 455.3505 [M − 60(CH_3_COOH) + H], 437.3442 [M − 18(H_2_O) − 60(CH_3_COOH) + H]^+^, 419.3207 [M − 60(CH_3_COOH) − 36(2H_2_O) + H]^+^ **MS^2^**: 515.3762→497.3629 [M − 18(H_2_O) + H]^+^, 479.3437 [M − 36(2H_2_O) + H]^+^, 437.3399 [M − 78(H_2_O + CH_3_COOH) + H]^+^ **MS^3^**: 497.3626→437.3391 [M − 18(H_2_O) − 60(CH_3_COOH) + H]^+^, 419.3360 [M − 18(H_2_O) − 78(H_2_O + CH_3_COOH) + H]^+^, 355.2645 [M − 18(H_2_O) − 142(side chain on D ring) + H]^+^, 295.2417 [M − 18(H_2_O) − 202(CH_3_COOH + side chain on D ring) + H]^+^, 437.3391→419.3295 [M − 78(H_2_O + CH_3_COOH) − 18(H_2_O) + H]^+^, 391.3359 [M − 78(H_2_O + CH_3_COOH) − 46(HCOOH) + H], 295.2419 [M − 78(H_2_O + CH_3_COOH) − 142(side chain on D ring) + H]^+^	3-*O*-Acetyl-16α-hydroxydehydrotra-metenolic acid [37]
**F17**	67.750	525.3584 527.3719	**MS^1^**: 525.3581 [M − H]^−^ **MS^2^**: 525.3603→509.3196 [M − H − 16(CH_4_)]^−^, 465.3379 [M − 60(CH_3_COOH) − H]^−^, 447.3200 [M − H − 78(H_2_O + CH_3_COOH)]^−^, 432.3020 [M − 93(CH_3_ + H_2_O + CH_3_COOH) − H]^−^	**MS^1^**: 527.3735 [M + H]^+^, 549.3522 [M + Na]^+^, 509.3624 [M − 18(H_2_O) + H]^+^, 481.3769 [M − 46(HCOOH) + H]^+^, 467.3539 [M − 60(CH_3_COOH) + H]^+^, 449.3400 [M − 78(H_2_O + CH_3_COOH) + H]^+^ **MS^2^**: 527.3735→509.3624 [M − 18(H_2_O) + H]^+^, 449.3465 [M − 78(H_2_O + CH_3_COOH) + H]^+^ **MS^3^**: 509.3624→491.3414 [M − 18(H_2_O) − 18(H_2_O) + H]^+^, 449.3399 [M − 18(H_2_O) − 60(CH_3_COOH) + H]^+^, 353.2453 [M − 18(H_2_O) − 156(side chain on D ring) + H]^+^, 293.2240 [M − 18(H_2_O) − 216(CH_3_COOH + side chain on D ring) + H]^+^, 449.3465→293.2249 [M − 78(H_2_O + CH_3_COOH) − 156(side chain on D ring) + H]^+^	3-*epi*-Dehydro-pachymic acid [37]
**F18**	76.858	587.3756 589.3883	**MS^1^**: 587.3756 [M − H]^−^ **MS^2^**: 587.3756→465.3296 [M − 122(HCO−Ar−OH) − H]^−^	**MS^1^**: 589.3883 [M + H]^+^, 611.3583 [M + Na]^+^, 571.3853 [M − 18(H_2_O) + H]^+^, 449.3413 [M − 18(H_2_O) − 122(HCO−Ar−OH) + H]^+^, 430.9047 [M − 36(2H_2_O) − 122(HCO−Ar−OH) + H]^+^, 406.2653 [M − 15(CH_3_) − 46(HCOOH) − 122(HCO−Ar−OH) + H]^+^ **MS^2^**: 589.3883→571.3813 [M − 18(H_2_O) + H]^+^ **MS^3^**: 571.3813→449.3361 [M − 18(H_2_O) − 122(HCO−Ar−OH) + H]^+^, 415.2603 [M − 18(H_2_O) − 156(side chain on D ring) + H], 403.0557 [M − 18(H_2_O) − 168(HCOOH+HCO−Ar−OH) + H]^+^, 293.2261 [M − 18(H_2_O) − 278(HCO−Ar−OH+ side chain on D ring) + H]^+^, 449.3413→419.0319 [M − 18(H_2_O) − 122(HCO−Ar−OH) − 30(2CH_3_) + H]^+^, 293.2260 [M − 18(H_2_O) − 122(HCO−Ar−OH) − 156(side chain on D ring) + H]^+^	3β-*p*-Hydroxybenzoyl-dehydrotumulosic acid [4]
**F19**	78.300	467.3152 469.3617	**MS^1^**: 467.3152 [M − H]^−^, 439.3557 [M − 28(CO) − H]^−^ **MS^2^**: 467.3152→451.3368 [M − 16(CH_4_) − H]^−^, 421.3552 [M − 46(HCOOH) − H]^−^, 292.9842 [M − 174(H_2_O + side chain on D ring) − H]^−^	**MS^1^**: 469.3617 [M + H]^+^, 451.3574 [M − 18(H_2_O) + H]^+^ **MS^2^**: 469.3617→451.3574 [M − 18(H_2_O) + H]^+^, 433.3207 [M − 36(2H_2_O) + H]^+^, 423.3302 [M − 46 (HCOOH) + H]^+^, 328.9961 [M − 140(RDA fragmentation of B ring) + H]^+^, 313.2356 [M − 156(side chain on D ring) + H]^+^ **MS^3^**: 451.3574→433.3207 [M − 18(H_2_O) − 18(H_2_O) + H]^+^, 311.2370 [M − 18(H_2_O) − 140(RDA fragmentation of B ring) + H]^+^, 295.2413 [M − 18(H_2_O) − 156(side chain on D ring) + H]^+^	Dehydroeburicoic acid [35]
**a1**	**26.285**	471.3478 473.3639	**MS^1^**: 471.3478 [M − H]^−^ **MS^2^**: 471.3478→409.3100 [M − 62(CH_4_ + HCOOH) − H]^−^	**MS^1^**: 473.3639 [M + H]^+^, 495.3469 [M + Na]^+^, 511.3243 [M + K]^+^, 457.3665 [M − 16(CH_4_) + H]^+^, 455.3527 [M − 18(H_2_O) + H]^+^, 437.3413 [M − 36(2H_2_O) + H]^+^, 429.2905 [M − 44(CO_2_) + H]^+^, 317.6939 [M − 156(CH_4_ + RDA fragmentation of B ring) + H]^+^ **MS^2^**: 473.3639→455.3515 [M − 18(H_2_O) + H]^+^, 437.3438 [M − 36(2H_2_O) + H]^+^ **MS^3^**: 455.3508→437.3415 [M − 18(H_2_O) − 18(H_2_O) + H]^+^, 313.2512 [M − 18(H_2_O) − 142(side chain on D ring) + H]^+^, 295.2432 [M − 18(H_2_O) − 160(H_2_O + side chain on D ring) + H]^+^, 437.3415→419.3394 [M − 36(2H_2_O) − 18(H_2_O) + H]^+^, 295.2422 [M − 36(2H_2_O) − 142(side chain on D ring) + H]^+^	16α-Hydroxy-trametenolic acid [34]
**a2**	**35.110**	485.3641 487.3779	**MS^1^**: 485.3641 [M − H]^−^ **MS^2^**: 485.3641→437.3421 [M − 48(3CH_4_) − H]^−^, 423.3261 [M − 62(CH_4_ + HCOOH) − H]^−^ **MS^3^**: 423.3261→407.3050 [M − 62(CH_4_ + HCOOH) − 6(CH_4_) − H]^−^	**MS^1^**: 487.3779 [M + H]^+^, 525.3343 [M + K]^+^, 509.3610 [M + Na]^+^, 469.3686 [M − 18(H_2_O) + H]^+^, 451.3542 [M − 36(2H_2_O) + H]^+^ **MS^2^**: 487.3779→469.3669 [M − 18(H_2_O) + H]^+^, 451.3542 [M − 36(2H_2_O) + H]^+^ **MS^3^**: 469.3669→451.3509 [M − 18(H_2_O) − 18(H_2_O) + H]^+^, 313.2502 [M − 18(H_2_O) − 156(side chain on D ring) + H]^+^, 295.2419 [M − 18(H_2_O) − 174(H_2_O+side chain on D ring) + H]^+^, 451.3542→433.3188 [M − 36(2H_2_O) − 18(H_2_O) + H]^+^, 309.2200 [M − 36(2H_2_O) − 142(side chain on D ring) + H]^+^, 295.2425 [M − 36(2H_2_O) − 156(H_2_O + side chain on D ring) + H]^+^	Tumulosic acid [32]
**a3**	**48.572**	483.3478 485.3613	**MS^1^**: 483.3478 [M − H]^−^ **MS^2^**: 483.3478→437.3382 [M − 46(HCOOH) − H]^−^, 421.3146 [M − 62(CH_4_ + HCOOH) −H]^−^, 405.3155 [M − 78(2CH_4_ + HCOOH) − H]^−^, 389.2812 [M − 94(3CH_4_ + HCOOH) − H]^−^, 369.2392 [M − 114(2CH_4_ + 2H_2_O + HCOOH) − H]^−^, 295.1952 [M − 188(2CH_4_ + side chain on D ring) − H]	**MS^1^**: 485.3613 [M + H]^+^, 507.3456 [M + Na]^+^, 467.3515 [M − 18(H_2_O) + H]^+^, 449.3399 [M − 36(2H_2_O) + H]^+^, 311.1682 [M − 174(H_2_O + side chain on D ring) + H]^+^ or [M − 174(2H_2_O+RDA fragmentation of B ring) + H]^+^, 301.1401 [M − 184(HCOOH+RDA fragmentation of B ring) + H]^+^ **MS^2^**: 485.3613→467.3506 [M − 18(H_2_O) + H]^+^, 449.3390 [M − 36(2H_2_O) + H]^+^, 311.2428 [M − 174(H_2_O + side chain on D ring) + H]^+^ **MS^3^**: 467.3506→449.3399 [M − 18(H_2_O) − 18(H_2_O) + H]^+^, 431.3310 [M − 18(H_2_O) − 36(2H_2_O) + H]^+^, 421.3452 [M − 18(H_2_O) − 46(HCOOH) + H]^+^, 311.2353 [M − 18(H_2_O) − 156(side chain on D ring) + H]^+^, 293.2247 [M − 18(H_2_O) − 174(H_2_O + side chain on D ring) + H]^+^, 449.3390→431.3378 [M − 36(2H_2_O) − 18(H_2_O) + H]^+^, 403.3292 [M − 36(2H_2_O) − 46(HCOOH) + H]^+^, 293.2258 [M − 36(2H_2_O) − 156(side chain on D ring) + H]^+^, 311.2428→293.2250 [M − 174(H_2_O + side chain on D ring) − 18(H_2_O) + H]^+^	Unknown
**a4**	**70.508**	527.3730 529.3897	**MS^1^**: 527.3730 [M − H]^−^ **MS^2^**: 527.3730→481.3658 [M − 46(HCOOH) − H]^−^, 465.3329 [M − 62(CH_4_ + HCOOH) − H]^−^, 431.2794 [M − 96(2H_2_O + CH_3_COOH) − H]^−^, 405.3045 [M − 122(CH_3_COOH + HCOOH + CH_4_) − H]^−^ **MS^3^**: 465.3329→405.3168 [M − 62(CH_4_ + HCOOH) − 60(CH_3_COOH) − H]^−^	**MS^1^**: 567.3456 [M + K]^+^, 551.3703 [M + Na]^+^, 529.3897 [M + H]^+^, 511.3759 [M − 18(H_2_O) + H]^+^, 493.3662 [M − 36(2H_2_O) + H]^+^, 469.3707 [M − 60(CH_3_COOH) + H]^+^, 451.3572 [M − 78(H_2_O + CH_3_COOH) + H]^+^ **MS^2^**: 529.3897→511.3764 [M − 18(H_2_O) + H]^+^, 451.3559 [M − 78(H_2_O + CH_3_COOH) + H]^+^ **MS^3^**: 511.3764→451.3555 [M − 18(H_2_O) − 60(CH_3_COOH) + H]^+^, 433.3480 [M − 18(H_2_O) − 78(H_2_O + CH_3_COOH) + H]^+^, 355.2589 [M − 18(H_2_O) − 156(side chain on D ring) + H]^+^, 295.2407 [M − 18(H_2_O) − 216(CH_3_COOH + side chain on D ring) + H]^+^, 451.3559→433.3485 [M − 78(H_2_O + CH_3_COOH) − 18(H_2_O) + H]^+^, 405.3540 [M − 78(H_2_O + CH_3_COOH) − 46(HCOOH) + H]^+^, 295.2412 [M − 78(H_2_O + CH_3_COOH) − 156(side chain on D ring) + H]^+^, 295.2407→280.2181 [M − 78(H_2_O + CH_3_COOH) − 156(side chain on D ring) − 15(CH_3_) + H]^+^	Pachymic acid [32]

**Table 3:** The factor loading matrix.

Peak No.	Six Principal Components ^a^					
	1	2	3	4	5	6
**F13**	0.855	0.027	−0.389	−0.055	0.071	−0.212
**F6**	0.848	0.165	−0.113	0.030	−0.367	0.183
**F4**	0.808	0.190	−0.015	−0.474	−0.006	0.130
**F7**	0.754	−0.186	0.255	0.214	0.214	−0.207
**F15**	0.744	−0.352	0.089	−0.293	−0.100	0.251
**F1**	0.648	0.133	−0.260	0.309	0.279	0.457
**F12**	0.596	0.529	−0.359	−0.396	0.258	−0.030
**F16**	0.559	−0.080	0.528	−0.289	−0.460	0.028
**F9**	0.549	−0.499	0.407	−0.127	0.263	−0.281
**F11**	0.535	−0.533	0.160	0.245	0.322	−0.349
**F8**	−0.310	0.810	−0.045	−0.240	0.329	0.042
**F10**	−0.244	0.768	0.174	0.190	−0.346	−0.279
**F17**	0.516	0.707	0.223	−0.075	−0.108	−0.303
**F5**	−0.124	0.688	0.232	−0.054	0.645	0.114
**F18**	0.186	0.604	0.349	−0.222	−0.114	0.050
**F19**	0.032	−0.208	0.767	0.186	0.280	0.446
**F3**	0.540	0.102	−0.630	0.494	0.133	−0.015
**F2**	0.383	0.397	0.069	0.614	−0.458	0.180
**F14**	0.267	0.500	0.474	0.505	0.168	−0.120

Extraction method: principal components. ^a^ The six components has extracted.

**Table 4:** The regions of origin of the 15 samples.

No.	Region	No.	Region
**S1**	Yuxi, Yunnan	**S9**	Xiangxi, Hunan
**S2**	Chuxiong, Yunnan	**S10**	Xinxiang, Henan
**S3**	Dali, Yunnan	**S11**	Yulin, Guangxi
**S4**	Lijiang, Yunnan	**S12**	Jinzhai, Anhui
**S5**	Luotian, Hubei	**S13**	Chengdu, Sichuan
**S6**	Shennongjia, Hubei	**S14**	Suining, Sichuan
**S7**	Yundu, Guizhou	**S15**	Yuexi, Anhui
**S8**	Fujian		
